# Thyroidectomy or Thiouracil?
*A Paper read to the Bristol Medico—Chirurgical Society on May 12th, 1948.


**Published:** 1948

**Authors:** A. Rendle Short


### THYROIDECTOMY OR THIOURACIL ?*

BY

A. RENDLE SHORT, M.D., B.S., B.Sc., F.R.C.S.

---

ON two previous occasions, in 1930[1] and in 1941,[2] I have had the honour of reading papers before the Bristol Medico-Chirurgical Society on the surgery of thyrotoxicosis. By that time, operation had become the routine treatment, except in quite mild or very severe cases, and a certain number of children or young women with primary thyrotoxicosis. In 1943, however, thiouracil was introduced by Astwood[3] in America, and a year or two later it came into general use in this country. Some physicians claim that it is as efficient as thyroidectomy in the treatment of toxic goitre, and without its hazards. This claim we are about to consider.

*Natural Prognosis of Thyrotoxicosis.* Before deciding upon the value of any treatment for any disease, it is well to know what course it is likely to follow with little or no special treatment. The best information we have is the experience of Hale White[4] in England, published in 1911, and of Rogers[5] in America, dated 1909. Their patients were treated by rest in bed, bromides, and belladonna.

TABLE I

NATURAL COURSE OF THYROTOXICOSIS

| | No. of Cases | Died in hospital | Followed | Cured per cent. | Better per cent. | No. better per cent. | Died since per cent. |
|---|---|---|---|---|---|---|---|
| Hale White | 216 | 11 | 102 | 57 | 23 | 5 | 15 |
| Rogers .. | 480 | — | 240 | 30 | 20 | 34 | 16 |

These figures are not exactly comparable with those of to-day. They refer not to thyrotoxicosis but to "exophthalmic goitre". There would not be many cases of toxic adenoma with a small or

* A Paper read to the Bristol Medico-Chirurgical Society on May 12th, 1948.

deep goitre and auricular fibrillation ; indeed, only patients with primary thyrotoxicosis and exophthalmos might be included. It may be taken for certain that Hale White's results would have been much worse if the thyrocardiac patients had not been omitted. The outlook is much better in cases of primary toxicosis than in those with secondary, and in the younger women than in the older.

*The Thiouracil Group of Drugs.*—Those in general use have been thiourea, thiouracil, methylthiouracil, and propylthiouracil. Thiourea has almost dropped out. Most of the published work relates to thiouracil. Probably the newer modifications are safer. These drugs act by reducing the internal secretion of thyroxin, but it is an important fact that they do not alter the pathological histology of the thyroid. They increase its vascularity, and it is more likely to swell than to diminish in size during their administration, at any rate in the early stages.

As a means of reducing toxicity the drugs are successful in the great majority of patients, but unfortunately they have their drawbacks. They may cause fever, skin rashes, or enlargement of the salivary glands. These complications are not serious. The real danger is agranulocytosis, leading to reduced resistance to infections and especially to septic sore throats. There have been a good many fatalities as a result. Statistics are appended showing that about one case in ten gets an unfavourable reaction of some kind, and about one in two hundred dies.

It is true that the methyl and propyl derivatives seem to be safer, and that prompt withdrawal of the drug and administration of penicillin may save patients whose lives were in danger.

TABLE II

COMPLICATIONS OF THIOURACIL TREATMENT

| | No. of Cases | Reactions per cent. | Agranulocytosis per cent. | Died per cent. |
|---|---|---|---|---|
| Van Winkle [6] .. | 5,724 | 13 | 2.5 | 0.3 |
| Fowler [7] .. .. | 1,573 | 13.7 | — | 0.57 |
| F. D. Moore [8] .. | 1,091 | 10 | — | 0.5 |
| Egerton Elliott [9] .. | 110 | — | 5 | 1 |
| Poate [10] .. .. | 84 | 10 | — | 0 |

If it is found that a patient can take thiouracil without mishap, what are the prospects that it will cure the thyrotoxicosis ? Reports by Himsworth[11] and colleagues, from University College Hospital, and from Eaton,[12] afford some evidence on this subject. It will be observed that fewer than half the cases are claimed as cures, even when the medical treatment has been prolonged for six to twenty-four months.

It will be clear from these observations, published by physicians who were interested in making a success of a line of medical treatment, that deliberate attempts to cure thyrotoxicosis with thiouracil are unwise, unless the patient is under careful observation in a hospital or nursing-home for the first month or two, and a leucocyte count is made week by week. The polymorphs sometimes drop very suddenly. When the disease has been brought under control and the dose has been reduced, these precautions can be relaxed somewhat.

Table III

RESULTS OF THIOURACIL TREATMENT

| Himsworth's Series* | No. of Cases |
|---|---|
| Patients | 91 |
| Came to operation | 12 |
| Reactions | 11 |
| Died | 2 |

Apparently cured (still have goitres) 40 per cent.

Of twenty-five cases, drug stopped after six to twenty-four months, seven relapsed.

Of six cases watched eighteen months, four relapsed.

*Eaton*. Of thirty-six cases treated in 1945, fourteen reported well and seventeen needing treatment.

*Thyroidectomy*.—Twenty years ago removal of the goitre for thyrotoxicosis was a serious proposition. My own mortality was 7 per cent. A good many patients were sent to the surgeon as a last resort, with huge highly vascular goitres, severe toxic symptoms or hearts worn out with fast driving and myocardial degeneration. These often died in fact, some of my cases died while waiting in hospital before operation. Nowadays these are seldom seen, and if seen are given long and careful preparatory treatment. Modern anæsthesia and modern surgical technique are definitely better than they were. Operation under a local anæsthetic saves lives in heavy-risk cases, but is not called for as a routine.

* This Paper was read before Professor Himsworth's (30.6.48), a note on which we hope to publish in the next issue.—*Ed.*

Although thyroid surgery is now remarkably safe, some physicians have lingering memories of the bad past and hold exaggerated ideas of its risks. Professor Tunbridge[13] of Leeds said in January, 1948, that even in good hands the operative mortality is still 10 per cent., and that only half the patients are cured. He is pessimistic also with regard to thiouracil treatment.

Whatever may be thought of the wisdom of attempting a long-term cure of thyrotoxicosis by means of drugs of this group, there is no doubt that they have a real value in preparing patients for the surgeon. Since using them F. H. Lahey[14] and his colleagues have lost only one case out of a thousand operated on.

*Personal Experience of Thyroidectomy.*—My own figures are given in the appended table. It should be mentioned that pressure goitres are included as well as toxic cases ; indeed, it is difficult sometimes to distinguish them. Not infrequently what would appear to be a pressure goitre is removed, but there is such a sharp thyrotoxic reaction that it becomes clear that there was toxic element already present. But in two-thirds of the cases here recorded there was no doubt about increased thyroid secretion.

The table shows three deaths since 1940. One of these was a patient with worn-out myocardium and auricular fibrillation. In another the cause of death was mysterious ; she lingered for a week before dying. Both these were before the days of thiouracil and might have been saved if it had been available. The third case was highly excitable and very toxic ; she had been well treated with thiouracil in hospital, but with little improvement.

TABLE IV

PERSONAL EXPERIENCE OF THYROIDECTOMY

| | No. of Cases | Mortality | |
|---|---|---|---|
| | | Deaths | per cent. |
| Total operations .. .. .. | 775 | | |
| 1937–1940 .. .. .. .. | 200 | 3 | 1.5 |
| 1941–1948 .. .. .. .. | 277 | 3 | 1.1 |
| Private patients and outlying hospitals .. .. .. .. | Last 198 | 0 | — |
| Fibrillating patients .. .. | 34 | 5 | 14.0 |
| Followed up .. .. .. | 154 | — | — |
| Well .. .. .. .. | 111 | — | — |
| Better .. .. .. .. | 36 | — | — |
| No better .. .. .. .. | 3 | — | — |
| Died since .. .. .. .. | 4 | — | — |

Deaths from hæmorrhage ought not to occur nowadays. Deaths from thyrotoxic reaction can nearly always be avoided by prolonged rest in bed, thiouracil and Lugol's iodine. Thiouracil makes the gland soft, friable and vascular. To overcome this drawback it should be stopped a week before operation, and Lugol's iodine given for two weeks along with thiouracil, and then for a week without, to reduce the vascularity. If the toxicosis is not severe, it is better to avoid thiouracil altogether.

The really risky cases are the thyrocardiacs, and Dunhill's dictum that there are a few cases which cannot be made safe for surgery remained true till a couple of years ago and may be true still. In the series reported, the mortality was 14 per cent. of thirty-four cases, but all these fatalities were before the advent of thiouracil, which might have enabled them to come through safely.

A follow-up of 154 cases for at least a year and usually for three or four years has been conducted. Those claimed as "cured" sometimes complain of having put on too much weight, but this is reduced when they become active. Of those described as "better" the commonest cause of the relative failure has been a certain amount of residual thyrotoxicosis from inadequate removal of goitre tissue, especially if the gland was scarcely at all enlarged. A few have become myxœdematous. Damage to the recurrent laryngeal nerve, and post-operative tetany, are seldom seen nowadays. I have seen two cases of what might be called subclinical tetany, in which the woman remained quite well until extra demands were made on her blood calcium. One of these was sent up because she had "fits" at four o'clock every morning. She was suckling a baby. The "fits" stopped at once when she was told to wean the child, to take calcium gluconate, and to keep a glass of milk by her bedside at night. The other patient was hustled into tetany by being starved for three days in preparation for a gynæcological operation. Both these episodes followed several years after the thyroidectomy.

In a few cases the persistent symptoms annoying the patient have been not serious, and difficult to explain, such as headaches or painful sensations in the scar: there was one case of exophthalmic ophthalmoplegia.

*Deductions.*—I offer the following tentative conclusions:

1. Thiouracil drugs control thyrotoxicosis, but one patient in ten suffers some kind of reaction. One in 200 so treated has died, usually from agranulocytosis. Hospitalization and frequent blood-counts are very desirable, at any rate for the first month.

2. These drugs are valuable as a means of pre-operative treatment in severe cases.

3. Long-term treatment with a view to cure without operation is less reliable than surgery. It may be suitable in children, when operation is refused, or in relapsed cases. Mild cases of thyrotoxicosis, well able to do their ordinary work, are usually better without it, but it may be given if they can be closely observed and frequent blood-counts taken.

4. Lugol's iodine is good treatment in the pre-operation period, or for the home treatment of mild cases, provided it is stopped after three weeks.

5. Even before the days of thiouracil, the operative mortality of thyroidectomy was reduced to between 1 and 2 per cent., and it has been further reduced now that these drugs are available. The great advantage of surgery is that it cures quickly. Drug treatment has to be kept up for years.

### REFERENCES

[1] A. Rendle Short, *Bristol Med. Chi. Jour.*, 1930, **47**, 185.
[2] Ibid., 1941, **58**, 1.
[3] E. B. Astwood, *J.A.M.A.*, 1943, **122**, 78.
[4] Hale White, *Guy's Hosp. Rep.*, 1911, **65**, 1.
[5] Rogers, *Ann. Surg.*, 1909, 1023.
[6] Van Winkle, *J.A.M.A.*, 1946, **130**, 343.
[7] E. F. Fowler, *Internat. Abstr. Surg.*, 1946, **83**, 313.
[8] F. D. Moore, *J.A.M.A.*, 1946, **130**, 243.
[9] Egerton Elliott, *Connecticut State Med. Jour.*, 1947, **7**, 508.
[10] H. R. G. Poate, *B.M.J.*, 1948, i, 808.
[11] H. P. Himsworth, M. E. Morgans, W. R. Trotter, *Lancet*, 1947, i, 241.
[12] J. C. Eaton, *Glasgow Med. Jour.*, 1947, **28**, 155.
[13] R. E. Tunbridge, *B.M.J. Sup.*, 1948, i, 36.
[14] F. H. Lahey, *Ann. Royal Coll. Surg.*, 1947, **6**, 277.

